# Effectiveness of interventions to improve rates of intravenous thrombolysis using behaviour change wheel functions: a systematic review and meta-analysis

**DOI:** 10.1186/s13012-020-01054-3

**Published:** 2020-11-04

**Authors:** Md Golam Hasnain, John R. Attia, Shahinoor Akter, Tabassum Rahman, Alix Hall, Isobel J. Hubbard, Christopher R. Levi, Christine L. Paul

**Affiliations:** 1 School of Medicine and Public Health (SMPH), University of Newcastle (UoN), Callaghan, New South Wales Australia; 2 Hunter Medical Research Institute (HMRI), New Lambton Heights, New South Wales Australia; 3 John Hunter Hospital, New Lambton Heights, New South Wales Australia; 4 Department of Anthropology, Jagannath University, Dhaka, Bangladesh; 5 Centre for Development, Economics and Sustainability, Monash University, Melbourne, Victoria Australia; 6 The Sydney Partnership for Health, Education, Research & Enterprise (SPHERE), Liverpool, New South Wales Australia

**Keywords:** Thrombolysis, Implementation, Intervention, Systematic review, Meta-analysis

## Abstract

**Background:**

Despite being one of the few evidence-based treatments for acute ischemic stroke, intravenous thrombolysis has low implementation rates—mainly due to a narrow therapeutic window and the health system changes required to deliver it within the recommended time. This systematic review and meta-analyses explores the differential effectiveness of intervention strategies aimed at improving the rates of intravenous thrombolysis based on the number and type of behaviour change wheel functions employed.

**Method:**

The following databases were searched: MEDLINE, EMBASE, PsycINFO, CINAHL and SCOPUS. Multiple authors independently completed study selection and extraction of data. The review included studies that investigated the effects of intervention strategies aimed at improving the rates of intravenous thrombolysis and/or onset-to-needle, onset-to-door and door-to-needle time for thrombolysis in patients with acute ischemic stroke. Interventions were coded according to the behaviour change wheel nomenclature. Study quality was assessed using the QualSyst scoring system for quantitative research methodologies. Random effects meta-analyses were used to examine effectiveness of interventions based on the behaviour change wheel model in improving rates of thrombolysis, while meta-regression was used to examine the association between the number of behaviour change wheel intervention strategies and intervention effectiveness.

**Results:**

Results from 77 studies were included. Five behaviour change wheel interventions, ‘Education’, ‘Persuasion’, ‘Training’, ‘Environmental restructuring’ and ‘Enablement’, were found to be employed among the included studies. Effects were similar across all intervention approaches regardless of type or number of behaviour change wheel-based strategies employed. High heterogeneity (*I*^2^ > 75%) was observed for all the pooled analyses. Publication bias was also identified.

**Conclusion:**

There was no evidence for preferring one type of behaviour change intervention strategy, nor for including multiple strategies in improving thrombolysis rates. However, the study results should be interpreted with caution, as they display high heterogeneity and publication bias.

Contribution to the literature This study is the first rigorous systematic review and meta-analysis, which evaluates the differential effect of intervention strategies aimed at improving rates of intravenous thrombolysis based on behaviour change wheel intervention function as an analytical framework.

This review illustrates that this field of research not only has high heterogeneity as previously known, but also provides new evidence of publication bias both within and across intervention types.

Although this study indicates that various strategies can be effective, it does not provide strong evidence supporting any specific strategy. Most studies do not have enough detail to unambiguously classify the intervention components.

## Background

Stroke causes 5.5 million deaths worldwide and requires substantial treatment and post-stroke care-related economic costs [1]. There are an estimated 80 million stroke survivors worldwide, with an increase in absolute numbers of disability-adjusted life years [1]. Acute ischemic stroke (AIS) refers to the most prevalent and disabling form of stroke [2]. Intravenous thrombolysis (IVT) is considered one of the mainstream therapies for AIS since its approval in 1996 by the United States Food and Drug Administration as a first-line treatment [3]. Despite substantial evidence for both the safety and cost-effectiveness of IVT, the implementation rate has remained persistently low [4]. Subsequently, over 50 published studies deploying a variety of trial designs have tested a variety of approaches to boost implementation rates. One major challenge for increasing IVT usage is reducing onset-to-needle time, the sum of the onset-to-door and the door-to-needle times. Several strategies to reduce door-to-needle time have already been tested and have achieved improvements in IVT rates. Delayed patient arrival at hospital remains, however, one of the major obstacles to better IVT implementation with many studies focusing on approaches aiming to reduce pre-hospital delay [5]. Several additional factors, relevant at both individual and organisational levels, have been identified as major rate-limiting factors for IVT implementation [6]. Patients’ and bystanders’ inability to recognise stroke symptoms and signs resulting in delayed response in seeking support from healthcare providers contributes to delayed hospital arrival [6]. Delays in stroke recognition by paramedics and hospital staff, delays in obtaining and interpreting brain imaging, inefficiencies in emergency stroke care, delays in obtaining treatment consent, an absence of decision support systems and protocols in emergency care facilities and physician perception of IVT efficacy and safety have also been identified as major factors that limit IVT implementation [5]. Consequently, several, often multi-faceted intervention strategies have been tested in efforts to improve the rates of IVT in AIS [7]. Such intervention strategies include telemedicine and ‘hub-spoke’ models, pre- and/or in-hospital notification, multi-disciplinary collaborative approaches and re-organisation of pre-hospital and hospital systems of care [4, 7].

Since the intervention strategies to date have used a variety of methods in various settings, we aim to perform a systematic review and meta-analysis to compare the effectiveness of the various forms of intervention strategies. Thus far, there are three published systematic reviews that have attempted to investigate these issues; however, each has limitations. The first systematic review, published in 2016, only included studies that met the Cochrane collaboration standards for practice and organisation of care study design criteria [8]. The second and third systematic reviews and meta-analyses were published in 2018 and 2019 [4, 7]. Both studies expressed the meta-analyses results based on various intervention approaches, but they did not use any specific operational definitions or theoretical approaches when grouping the studies in the analyses [4, 7]. Moreover, neither study explored publication bias when describing group-based results [4, 7], despite the importance of this information to data interpretation.

Theory-based analysis of interventions is recommended when investigating the effect of a specific intervention strategy and aiding in the specification of a potentially active process of care [9]. For example, behaviour-targeted theories can be utilised to define the components of implementation interventions [10]. Multi-level, multi-disciplinary testing and decision-making processes are needed to identify a patient’s eligibility for IVT [11]. Therefore, increasing rates of IVT in AIS are considered an example where multiple factors could be critical to the design of targeted intervention strategies [8]. Conceptual frameworks, such as the behaviour change wheel (BCW), can be useful in defining the range of factors (e.g. training) that need to be addressed to effect complex change [12]. BCW is a behavioural framework which has at its centre the Capability, Opportunity, Motivation-Behaviour (COM-B) theory. As described by Nilsen et al. 2015 [13], the COM-B is an implementation theory which is useful for providing an understanding or an explanation of aspects of implementation. The overarching BCW framework specifies intervention functions (e.g. education, persuasion, training), which can be used to develop intervention content (i.e. a process model) and guide evaluation of an implementation intervention (e.g. an evaluation framework) [13]. Therefore, the BCW framework provides a useful structure for categorising and understanding the content of previous interventions and considering their potential implications; particularly in the context of literature where in-depth detail about intervention content is not commonly provided. In the context of stroke care, considering the existing literature in terms of intervention functions rather than solely pooling all implementation studies has the potential to provide additional understanding regarding how to successfully implement a complex multi-component practice like IVT in a given healthcare organisation. Thus, an evaluation of the interventions aimed at improving the rates of IVT in AIS including coding them based on BCW intervention function could assist to identify more clearly the approaches which might be associated with higher rates of IVT implementation.

The core aspect of the BCW framework described by Michie et al. consists of a ‘behaviour system’ that includes three key elements: capability, opportunity and motivation. This core aspect is surrounded by nine intervention functions and then by seven policy categories [12]. The intervention functions help to identify the gaps and highlight the areas that need intervention. For example, the intervention functions were used to characterise interventions related to smoking cessation and reducing obesity [12]. The functions can also be used to contextualise already implemented interventions and to lead to more efficient design of effective interventions. To date, no studies have used the BCW classification as a framework for examining the intervention strategies aimed at improving the rates of IVT. To synthesise the results of studies which tested the effect of intervention strategies aimed at improving the rates of IVT in AIS, we will use the BCW nomenclature as the analytical context with the following primary and secondary objectives:

❖ Primary objective: to explore the differential effectiveness of the intervention strategies aimed at improving the rates of IVT based on the number and type of BCW intervention functions employed.

❖ Secondary objective: to describe the number and type of BCW intervention functions employed in intervention strategies aimed at improving the rates of IVT.

## Methods

This systematic review follows the Preferred Reporting Items for Systematic reviews and Meta-analyses (PRISMA) statement guidelines [14]. The PRISMA statement is provided in Supplementary file 1. This systematic review was not registered.

### Searches

MEDLINE, EMBASE, PsycINFO, CINAHL and SCOPUS databases were searched for articles published from January 1996 to December 2018 in English. We also checked the reference lists of included articles and existing systematic reviews for relevant studies. The search dates were selected to coincide with the 1996 approval and release of the first thrombolysis guideline for AIS [15].

We followed the search strategy described by Paul et al. 2016 [8] while selecting the search terms. The search terms are a combination of keyword searches: ‘Tissue plasminogen activator’ OR ‘tPA’ OR ‘rtPA’ OR ‘Alteplase’ OR ‘Thrombolysis’ AND ‘Stroke’ OR ‘Ischemic stroke’ OR ‘Brain ischemia’ OR ‘Middle cerebral artery infarction’ OR ‘Cerebrovascular disorder’ OR ‘Cerebrovascular accident’ OR ‘CVA’ OR ‘Cerebral stroke’ OR ‘Cerebral accident’ OR ‘Cerebral infarction’ OR ‘Cerebral apoplexy’ OR ‘Cerebrovascular apoplexy’. We used available MeSH headings; otherwise, a ‘multi-purpose; mp.’ field search was conducted. One senior librarian reviewed the final search strategy. The detailed search strategy is provided in Supplementary file 2.

### Inclusion and exclusion criteria

This systematic review included the following: quantitative studies that investigated the effect of interventions to improve the rates of IVT and/or onset-to-needle, onset-to-door and door-to-needle time for IVT in patients with AIS; studies that reported the rates of IVT utilisation and/or onset-to-door and/or door-to-needle time for thrombolysis as their primary outcome; studies that reported the number of patients with AIS who received IVT and the total number of suspected stroke and/or confirmed stroke and/or confirmed ischemic stroke patients and/or the total number of eligible patients for IVT; randomised controlled trials and cluster-randomised trials; non-randomised studies, such as uncontrolled before-after studies; parallel group trials; and observational studies including cohort, case-control and cross-sectional studies. The systematic review included a wide range of study designs to identify the possible reasons and factors behind the systematic review result [16]. Inclusion was also limited to original human research studies.

The review excluded studies that reported only haemorrhagic stroke or transient ischemic attack and those reporting rates of thrombolysis other than IVT. Key exclusion and inclusion points are summarised in Supplementary file 3.

### Outcome measures

We used IVT rates as our outcome of interest. The numerator was the total number of patients who received IVT, and the denominator was the total number of suspected stroke, confirmed stroke, confirmed ischemic stroke or IVT-eligible patients.

### Potential effect modifiers and reasons for heterogeneity

Outcome measures were grouped as follows, to explain some of the heterogeneity across the studies:

❖ Hospital factors addressed by the intervention (pre-hospital, in-hospital or both pre- and in-hospital)

❖ Denominator used (suspected stroke, confirmed stroke, confirmed ischemic stroke or IVT-eligible patients).

❖ Epidemiological design of the study (uncontrolled before-and-after, parallel group trial or randomised controlled trial).

The number of BCW intervention functions employed was included as a covariate in a meta-regression to examine the association between number of BCW intervention functions employed and intervention effectiveness and to explain the heterogeneity between the studies.

### Study quality assessment

Three reviewers assessed the quality of the studies. The principal reviewer (MGH) independently assessed the methodological quality of the final articles using the QualSyst scoring system for quantitative research methodologies [17]. The total number of included studies was then divided between two independent reviewers (SA and TR), who also assessed the quality of the studies using the same scoring system. Joint discussion between all three reviewers resolved any disagreements. Studies were scored depending on whether they fully met the criteria (2 points), partially met the criteria (1 point) or did not meet the criteria at all (0 points). Quantitative studies were scored against 14 criteria. A criterion for ‘evidence of ethical approach’ was added to the QualSyst scoring, resulting in a maximum total possible score of 22 for qualitative designs and 30 for quantitative designs [18].

### Study selection

All reviewers reviewed the titles and abstracts of the last 500 search results to identify abstracts that would potentially meet the inclusion criteria. There was > 95% agreement between reviewers. After removing duplicates, the principal reviewer reviewed all titles and abstracts and the full text of each non-rejected article to arrive at final inclusion determinations. The other two reviewers concurrently and independently reviewed half of the titles and abstracts and the full texts of non-rejected articles each and then compared their determinations to those made by the principal reviewer. The study selection process is described in more detail in Supplementary file 4 and Supplementary file 5

### Data extraction and coding strategy

The principal reviewer performed data extraction for all articles independently identified as ‘included’. A data extraction form was developed following the format used in the *Cochrane Handbook for Systemic Reviews of Interventions* [19]*.* This process recorded the following information: country, setting, publication year, intervention duration, study type based on epidemiological design, description of the intervention and outcome. The outcome used the total number of patients receiving IVT as the numerator and the total number of suspected stroke, confirmed stroke, confirmed ischemic stroke or IVT-eligible patients as the denominator. To ensure consistency, a data extraction form was pilot-tested on a 5% subset of the included full-text studies.

The interventions in the included studies were coded according to BCW framework intervention functions criteria mentioned in Michie et al [10].. Operational definitions for the included intervention functions have been described in Table 1. Three reviewers were involved in the process of coding. The principal author (MGH) independently identified the BCW intervention functions addressed through the interventions of the 77 studies. The total number of included studies was then divided between another two co-authors (SA and TR), who also identified the BCW intervention functions addressed through the interventions of the 38 and 39 studies respectively. The discrepancy between MGH and SA was 10.5% (4 out of 38) and between MGH and TR was 7.7% (3 out of 39). Joint discussion between all three reviewers resolved any disagreements. All coding discrepancies were then reviewed and finalised by the senior author (CP).

**Table 1:** Operational definitions for the assessed intervention components

Intervention component	Definition
Education	Providing systematic education or instruction to increase knowledge or understanding on stroke via face-to-face or online educational session, or by providing print or online educational materials to the health professionals from any level or community members.
Persuasion	Improve communication to stimulate the treatment process in patients with stroke.
Training	Providing systematic training to the community members or health professionals from any level to improve their skills in identifying suspected stroke cases. AND OR Providing systematic training to the health professionals from any level to improve their skills in diagnosing and treating stroke cases.
Environmental Restructuring	Restructuring, reorganising or rearranging individual, social or organisational context to promote the usage of thrombolysis in stroke.
Enablement	Increasing resources such or reducing obstacles to increase capability or opportunity at the individual level, e.g. health care staff or organisational level, e.g. hospital to promote the process and quality of stroke care.

Studies were categorised based on the number and type of BCW interventions employed. Specifically, studies were categorised based on:

❖ The number of BCW components implemented as part of the intervention, with categories including 1, 2, 3 or > 3.

❖ Whether one of the following components were included as part of the intervention: education, persuasion, incentivisation, coercion, training, restriction, environmental restructuring, modelling and enablement.

### Statistical analyses

Separate random effects meta-analyses by type of BCW strategy (i.e. education, persuasion, training, environmental, restructuring and enablement) and by number of BCW strategies implemented (i.e. one, two, three and more than 3) were conducted to assess the effectiveness of the interventions on improving IVT rates (primary objective). For each meta-analysis, a pooled odds ratio (OR) with a 95% confidence interval (CI) was calculated using the *metan* command in Stata. In addition, forest plots were used to show the effect sizes, to assess possible heterogeneity and identify potential outliers. Statistical heterogeneity was assessed by chi-square and *I*^2^ statistics. Publication bias was assessed by testing the asymmetry of funnel and contour-enhanced funnel plots, which were created using the *metafunnel* and *confunnel* commands in Stata, and via Egger’s test, which was conducted using the *metabias* command which was used in Stata. The impact of potential outliers on the results was assessed by conducting sensitivity analyses whereby any identified outliers were removed and the analysis re-run. Descriptive analyses were used to report the number and type of BCW framework interventions used as part of the study intervention (secondary objective). Finally, to determine if the number of BCW intervention strategies used were associated with intervention effectiveness, meta-regression was conducted using the *metareg* command in Stata. Stata (StataCorp LLC, College Station, Texas, USA) version 14 was used to conduct all analyses.

## Results

### Description of studies

**Fig. 1:**
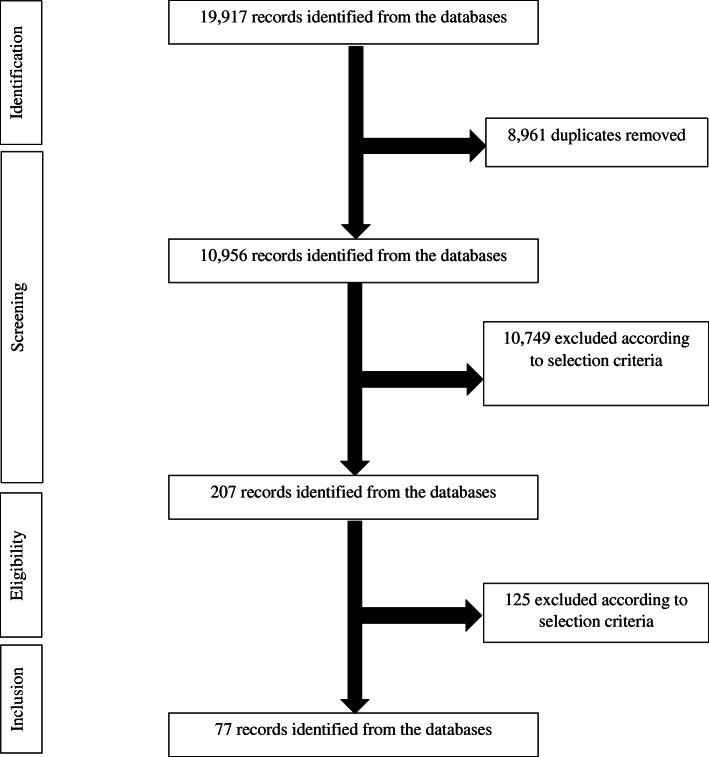
Flow diagram of studies included in the systematic review

A total of 19,917 titles were screened following database searches and hand searching of bibliographies, and 8961 titles were excluded (Fig. 1). The search hits of all databases are showed in Supplement 2. The remaining 10,749 abstracts were reviewed. A total of 207 articles were included for full-text data review, and 77 were selected based on the inclusion and exclusion criteria. BCW classifications were coded for all interventions described in the eligible manuscripts and then included in the meta-analyses.

### Characteristics of studies

The 77 studies included in the meta-analyses represent 40,614 IVT cases, and their general characteristics are shown in Table 1. All were published between 2002 and 2018. There were 29 (38%) studies from European countries, 26 (34%) from North American countries, 17 (22%) from Asian countries, four (5%) from Australia and one (1%) from South America. In the methods used, 58% (45/77) were uncontrolled before-and-after, 29% (22/77) were parallel group trial and 13% (10/77) were randomised controlled in design. For the factors the interventions addressed, 35 (45%) addressed in-hospital factors, 33 (43%) addressed pre- and in-hospital factors and nine (12%) addressed pre-hospital factors only.

### Quality of studies

The quality scores across studies were normally distributed with a mean of 78 and SD of 11, as shown in Table 2. The highest mean score of 97 (SD 7) was observed in the randomised controlled trial group, followed by similar scores of 76 (SD 7) and 74 (SD 9) for the parallel group trial and the uncontrolled before-and-after study group, respectively.

**Table 2:** Summary of study characteristics and outcome measures

First author and publication year	Study information				BCW intervention functions employed		Number received IVT		Total number of patients		% received IVT	
	Country	Addressed factor	Study design	Quality score (%)	Type^a^	Number	Intervention	Control	Intervention	Control	Intervention	Control
Behrens et al. 2002 [20]	Germany	Pre- and in-hospital	Uncontrolled before and after	63	1,5,9	3	9	1	86	57	10	2
Morgenstern et al. 2003 [21]	USA	Pre- and in-hospital	Parallel group trial	89	1,5,7,8,9	5	9	1	80	70	11	1
Lattimore et al. 2003 [22]	USA	In-hospital	Uncontrolled before and after	50	1,5,7,9	4	44	3	420	200	10	2
Belvis et al. 2005 [23]	Spain	Pre- and in-hospital	Parallel group trial	67	2,	1	7	8	37	181	19	4
Wojner-Alexandrov et al. 2005 [24]	USA	Pre- and in-hospital	Uncontrolled before and after	75	1,2,5,9,7	4	64	21	709	192	9	11
Ickenstein et al. 2005 [25]	Germany	In-hospital	Uncontrolled before and after	67	2,5,9,1,7	5	45	10	164	155	27	6
Nam et al. 2007 [26]	South Korea	In-hospital	Uncontrolled before and after	67	2,7,9	3	25	14	213	529	12	3
Demaerschalk et al. 2008 [27]	USA	Pre- and in-hospital	Uncontrolled before and after	71	1,9	2	320	4	1800	1454	18	0
Abdullah et al. 2008 [28]	USA	Pre- and in-hospital	Parallel group trial	79	2,	1	18	16	44	74	41	22
Quain et al. 2008 [29]	Australia	Pre- and in-hospital	Uncontrolled before and after	88	2,5,7,9	4	30	5	140	107	21	5
Gladstone et al. 2009 [30]	Canada	Pre- and in-hospital	Uncontrolled before and after	58	2,7,9	3	30	7	128	74	23	9
Pedrogosa et al. 2009 [31]	Spain	In-hospital	Uncontrolled before and after	71	2,9	2	19	9	198	201	10	4
Chenkin et al. 2009 [32]	Canada	Pre- and in-hospital	Uncontrolled before and after	79	5,2,9	3	56	18	554	307	10	6
Muller-Nordhorn et al. 2009 [33]	Germany	Pre-hospital	Randomised controlled trial	100	1,5	2	17	13	741	647	2	2
Kim et al. 2009 [34]	South Korea	Pre- and in-hospital	Uncontrolled before and after	71	2,9,7	3	47	44	328	678	14	6
Heo et al. 2010 [35]	South Korea	In-hospital	Uncontrolled before and after	71	7,9,2	3	312	199	5404	5798	6	3
Dharmasaroja et al. 2010 [36]	Thailand	In-hospital	Uncontrolled before and after	67	1,2,5,9	4	110	14	406	170	27	8
Bae et al. 2010 [37]	South Korea	Pre- and in-hospital	Parallel group trial	79	2,	1	33	18	55	47	60	38
Sung et al. 2011 [38]	Taiwan	In-hospital	Uncontrolled before and after	75	9,	1	21	40	139	338	15	12
Reiner-Deitemyer et al. 2011 [39]	Austria	Pre-hospital	Parallel group trial	63	9,	1	224	1062	898	13,731	25	8
Hoegerl et al. 2011 [40]	USA	In-hospital	Uncontrolled before and after	79	1,7,9	3	12	4	101	132	12	3
Etgen et al. 2011 [41]	Germany	In-hospital	Uncontrolled before and after	71	5,7,9,2	4	95	24	742	500	13	5
Walter et al. 2011 [42]	Germany	In-hospital	Uncontrolled before and after	79	9,7	2	16	32	78	120	21	27
Driks et al. 2011 [43]	Netherland	In-hospital	Randomised controlled trial	100	1,7,9	3	391	305	2990	2525	13	12
Addo et al. 2012 [44]	UK	Pre-hospital	Uncontrolled before and after	88	1,	1	45	55	274	326	16	17
Walter et al. 2012 [42]	Germany	Pre-hospital	Randomised controlled trial	100	9,7	2	12	8	53	47	23	17
O'Brien et al. 2012 [45]	Australia	Pre- and in-hospital	Uncontrolled before and after	67	1,2	2	22	5	115	67	19	7
Berglund et al. 2012 [46]	Sweden	Pre- and in-hospital	Randomised controlled trial	81	2,9	2	60	24	488	454	12	5
Prabhakaran et al. 2012 [47]	USA	In-hospital	Parallel group trial	71	9,	1	3335	732	58,512	61,027	6	1
Bhatt et al. 2012 [48]	USA	In-hospital	Parallel group trial	75	9,	1	47	60	484	789	10	8
Scott et al. 2013 [49]	USA	In-hospital	Randomised controlled trial	100	1,2,9	3	235	194	8419	9222	3	2
Meretoja et al. 2013 [50]	Australia	In-hospital	Uncontrolled before and after	79	2,7,9	3	47	82	324	468	15	18
McKinney et al. 2013 [51]	USA	Pre- and in-hospital	Parallel group trial	88	2,	1	31	17	114	115	27	15
Amorim et al. 2013 [52]	USA	In-hospital	Uncontrolled before and after	67	9,2	2	113	26	1669	919	7	3
Nolte et al. 2013 [53]	Germany	In-hospital	Uncontrolled before and after	79	2,	1	51	14	64	36	80	39
Prabhakaran et al. 2013 [47]	USA	Pre- and in-hospital	Uncontrolled before and after	79	2,	1	64	22	787	719	8	3
Schaik et al. 2014 [54]	Netherland	Pre- and in-hospital	Uncontrolled before and after	83	2,7,9	3	185	41	951	828	19	5
Fonarow et al. 2014 [55]	USA	In-hospital	Uncontrolled before and after	79	2,7,9	3	3552	1557	43,850	27,319	8	6
Ebinger et al. 2014 [56]	Germany	Pre-hospital	Randomised controlled trial	100	9,7	2	310	220	1070	1041	29	21
Minnerup et al. 2014 [57]	Germany	Pre- and in-hospital	Parallel group trial	63	9,	1	13,240	622	71,349	22,549	19	3
Camerlingo et al. 2014 [58]	Italy	Pre- and in-hospital	Uncontrolled before and after	83	1,5,9	3	23	8	401	376	6	2
Ruff et al. 2014 [59]	USA	Pre- and in-hospital	Uncontrolled before and after	75	2,7,9	3	142	116	925	1413	15	8
Hesselfeldt et al. 2014 [60]	Denmark	Pre-hospital	Parallel group trial	75	9,	1	22	87	65	265	34	33
Chen et al. 2014 [61]	Taiwan	In-hospital	Uncontrolled before and after	88	2,	1	216	91	2512	3500	9	3
Martinez-Sanchez et al. 2014 [62]	Spain	In-hospital	Uncontrolled before and after	83	9,2	2	18	12	225	259	8	5
Ragoschke-Schumm et al. 2015 [63]	UK	In-hospital	Parallel group trial	75	7,	1	66	49	174	81	38	60
Willeit et al. 2015 [64]	Austria	Pre- and in-hospital	Uncontrolled before and after	75	2,7	2	160	82	1238	1237	13	7
Kim et al. 2015 [65]	South Korea	In-hospital	Uncontrolled before and after	75	2,	1	202	44	341	132	59	33
Baldin et al. 2015 [66]	Australia	In-hospital	Uncontrolled before and after	79	9,2	2	16	10	62	58	26	17
Moran et al. 2016 [67]	Hawaii	In-hospital	Uncontrolled before and after	75	9, or 7	1	122	44	388	151	31	29
Busby et al. 2016 [68]	USA	Pre- and in-hospital	Uncontrolled before and after	75	2,	1	52	41	397	414	13	10
Choi et al. 2016 [69]	South Korea	In-hospital	Uncontrolled before and after	58	7,9	2	118	111	2078	2172	6	5
Nishijima et al. 2016 [70]	Japan	Pre-hospital	Uncontrolled before and after	71	1,	1	36	41	600	544	6	8
Kim et al. 2016 [71]	South Korea	Pre- and in-hospital	Parallel group trial	75	2,	1	215	59	306	136	70	43
Prabhakaran et al. 2016 [72]	USA	In-hospital	Parallel group trial	75	1,3,7,9	4	766	1626	10,314	15,261	7	11
Hubert et al. 2016 [73]	Finland	In-hospital	Parallel group trial	83	9,2	2	1779	912	11,589	3387	15	27
Itrat et al. 2016 [74]	USA	Pre-hospital	Parallel group trial	83	9,7	2	16	13	110	56	15	23
Denti et al. 2017 [75]	Italy	Pre-hospital	Randomised controlled trial	100	1,	1	145	156	649	503	22	31
Zaidi et al. 2017 [76]	USA	Pre- and in-hospital	Parallel group trial	79	2,5,7	3	28	18	109	142	26	13
Heikkila et al. 2019 [77]	USA	Pre- and in-hospital	Uncontrolled before and after	71	9,	1	51	124	355	1581	14	8
Hsiao et al. 2018 [78]	Taiwan	In-hospital	Parallel group trial	75	2,	1	40	18	254	118	16	15
Carvalho et al. 2018 [79]	Brazil	In-hospital	Uncontrolled before and after	75	5,7	2	28	13	154	61	18	21
Gurav et al. 2018 [80]	India	In-hospital	Uncontrolled before and after	75	2,	1	65	44	610	695	11	6
Tan et al. 2018 [81]	Sweden	Pre- and in-hospital	Uncontrolled before and after	75	2,7	2	281	129	826	416	34	31
Haesebaert et al. 2018 [82]	France	Pre- and in-hospital	Randomised controlled trial	100	1,5,7	3	140	114	363	328	39	35
Nguyen-Huynh et al. 2018 [83]	USA	Pre- and in-hospital	Uncontrolled before and after	88	2,7,9	3	557	310	3168	2375	18	13
Cone et al. 2018 [84]	USA	Pre- and in-hospital	Uncontrolled before and after	83	2,	1	94	181	219	316	43	57
Sadeghi-Hokmabadi et al. 2018 [85]	Iran	Pre- and in-hospital	Parallel group trial	79	2,	1	131	115	180	194	73	59
Meyer et al. 2008 [86]	USA	In-hospital	Randomised controlled trial	89	9,2	2	31	28	111	111	28	25
de Luca et al. 2009 [87]	Italy	In-hospital	Randomised controlled trial	100	5,9	2	15	2	434	328	3	1
Muller-Barna et al. 2014 [88]	Germany	In-hospital	Uncontrolled before and after	83	9,2	2	655	63	4409	2466	15	3
Vidale et al. 2016 [89]	Italy	Pre- and in-hospital	Parallel group trial	75	2,5,7	3	37	14	610	424	6	3
Nardetto et al. 2016 [90]	Italy	In-hospital	Parallel group trial	71	9,2	2	25	106	75	318	33	33
Hsieh et al. 2016 [91]	Taiwan	Pre- and in-hospital	Parallel group trial	79	2,	1	144	25	727	201	20	12
Jeon et al. 2017 [92]	South Korea	Pre- and in-hospital	Uncontrolled before and after	83	2,7	2	34	141	297	2012	11	7
Zhou et al. 2017 [93]	China	In-hospital	Parallel group trial	71	9,	1	231	88	712	689	32	13
Henry-Morrow et al. 2017 [94]	USA	Pre- and in-hospital	Uncontrolled before and after	50	1,	1	16	8	82	38	20	21

^a^Coding of BCW intervention function type: Education = 1, Persuasion = 2, Incentivisation = 3, Coercion = 4, Training = 5, Restriction = 6, Environmental restructuring = 7, Modelling = 8, Enablement = 9

### Description on behaviour change wheel categories

Twenty nine (38%) studies implemented one BCW component, 22 (29%) implemented two components, 18 (23%) implemented three components and 8 (10%) implemented more than 3 components. Of the types of BCW interventions included, 47 (61%) studies included at least enablement, 46 (60%) included at least persuasion, 31 (40%) at least environmental restructuring, 19 (25%) at least education and 16 (21%) at least training.

### Outcome measures

**Fig. 2:**
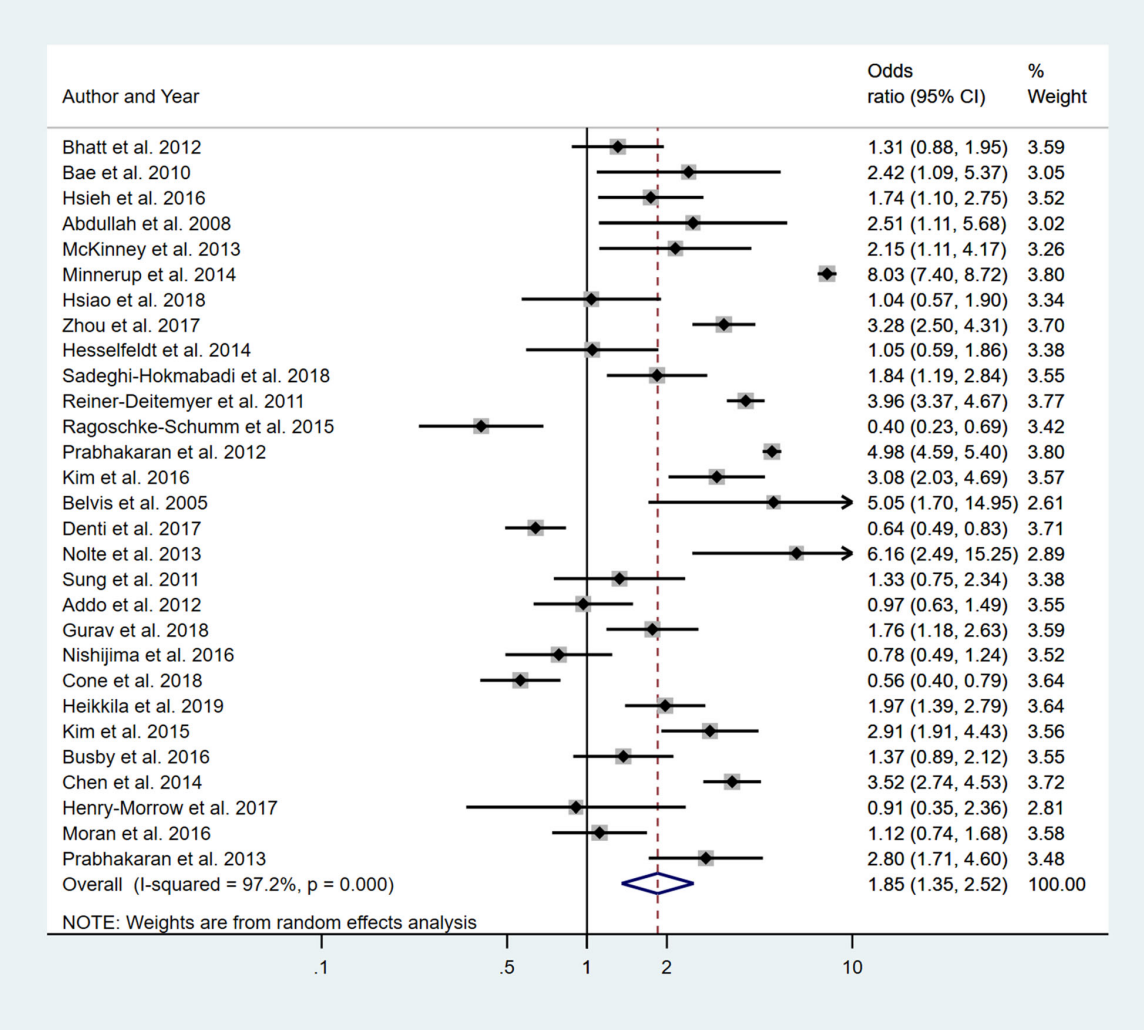
Pooled odds ratio of the intervention effectiveness in the studies that included only one BCW intervention

**Fig. 3:**
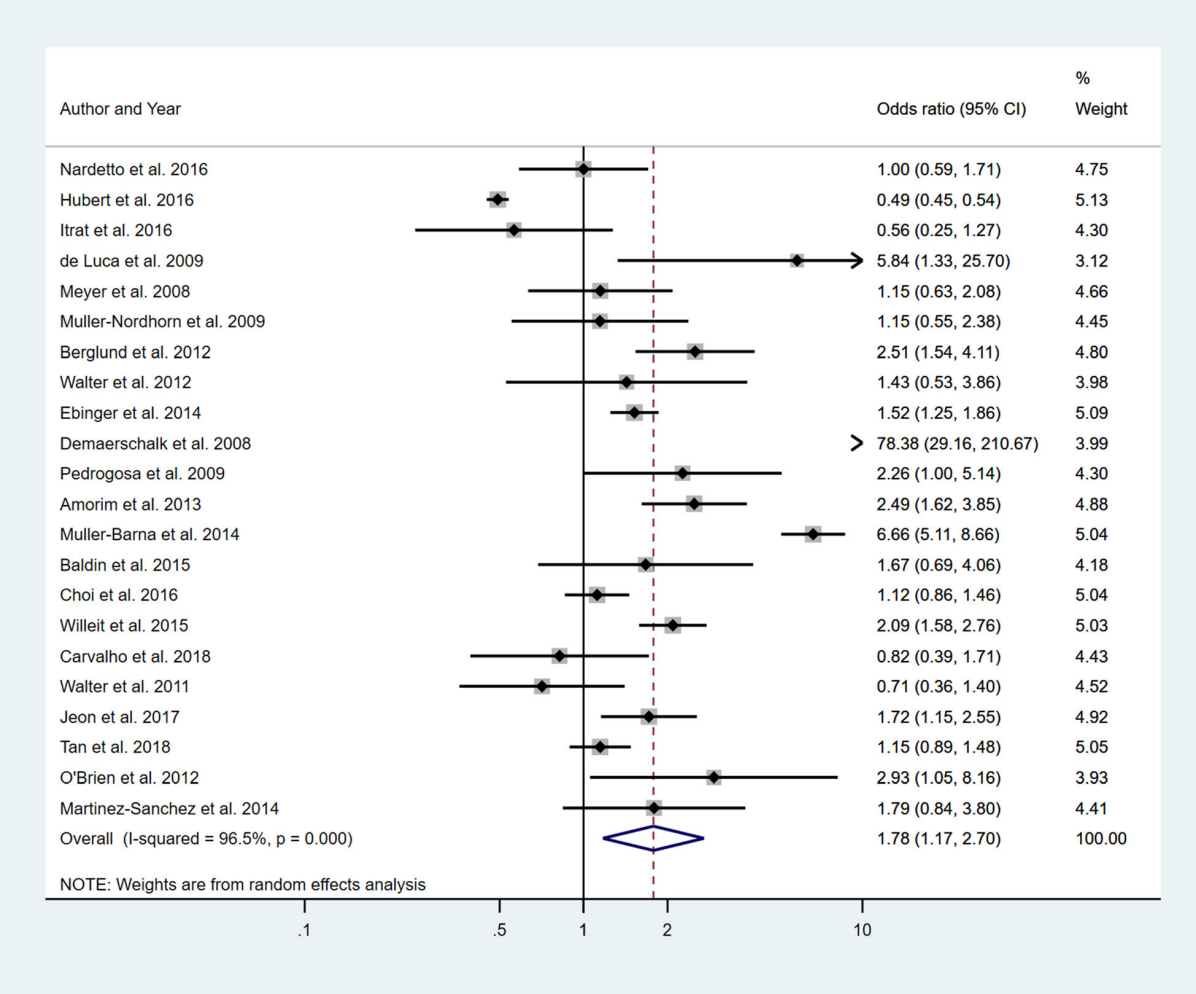
Pooled odds ratio of the intervention effectiveness in the studies that included two BCW intervention functions

**Fig. 4:**
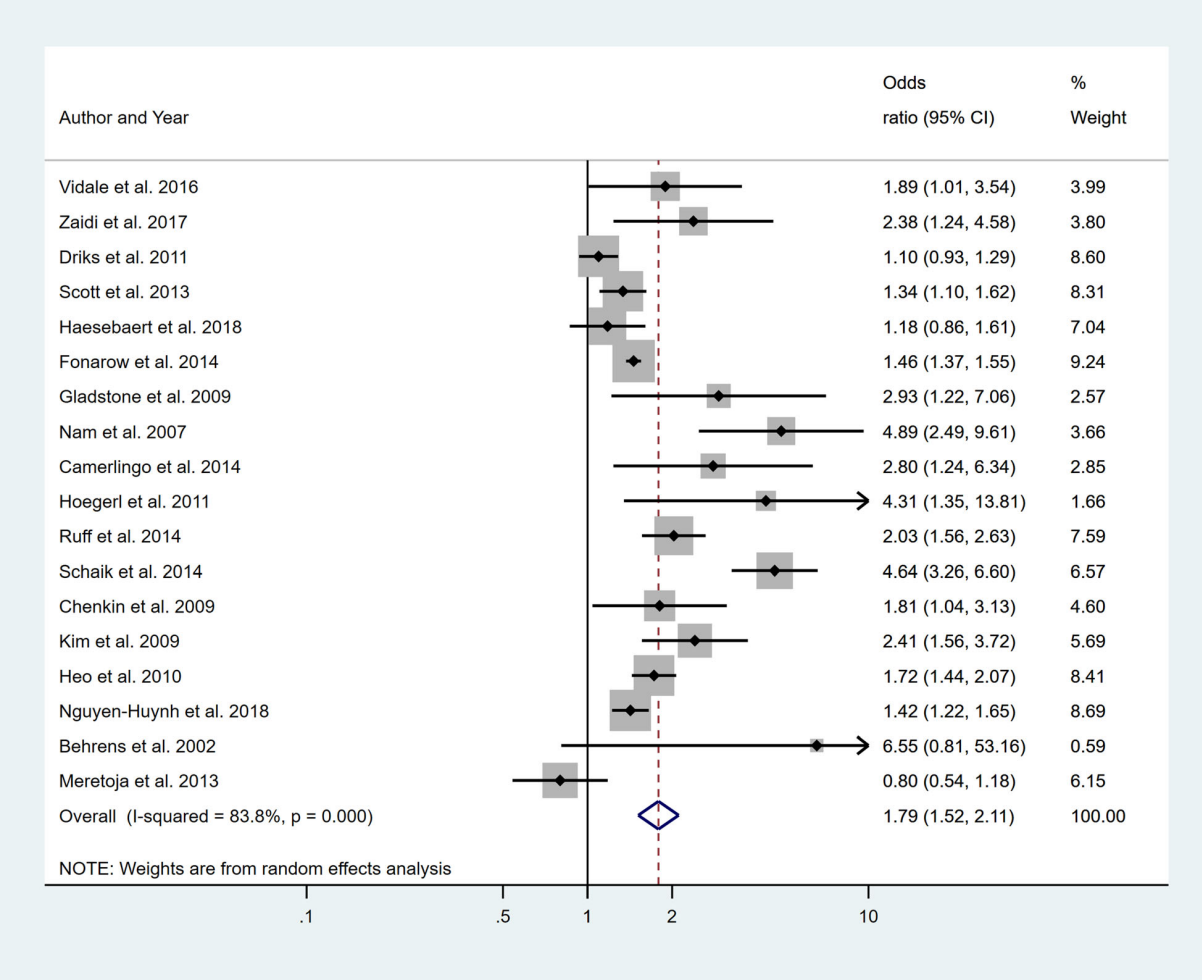
Pooled odds ratio of the intervention effectiveness in the studies that included three BCW intervention functions

**Fig. 5:**
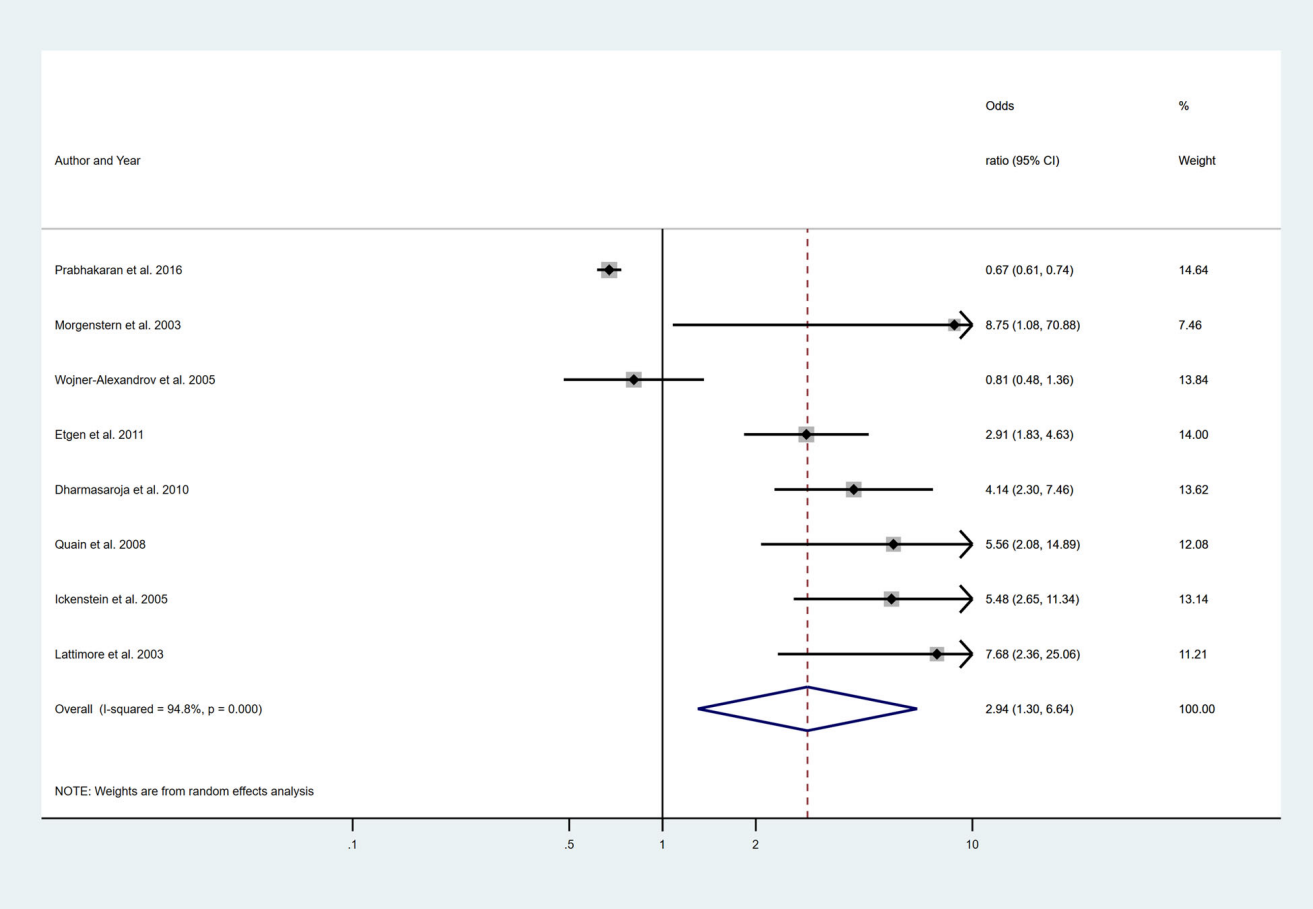
Pooled odds ratio of the intervention effectiveness in the studies that included four to five BCW intervention functions

Four separate meta-analyses were conducted to assess the effect of incorporating one, two, three or more BCW intervention types (Fig. 2, 3, 4 and 5). All four analyses found significant overall improvements in rates of IVT delivery, with odds ratio of between 1.78 to 2.94, with largely overlapping confidence intervals. High heterogeneity was seen in all meta-analyses (*I*^2^ range 84 to 97%). Sub-group analyses based on the hospital factors addressed by the intervention, the denominator used and epidemiological design of the study indicated that heterogeneity (*I*^2^) still ranged from moderate to high. A sensitivity analysis that excluded the results of an outlier study (Demaerschalk et al. [27]) did not result in any substantial change in the results or conclusions.

**Fig. 6:**
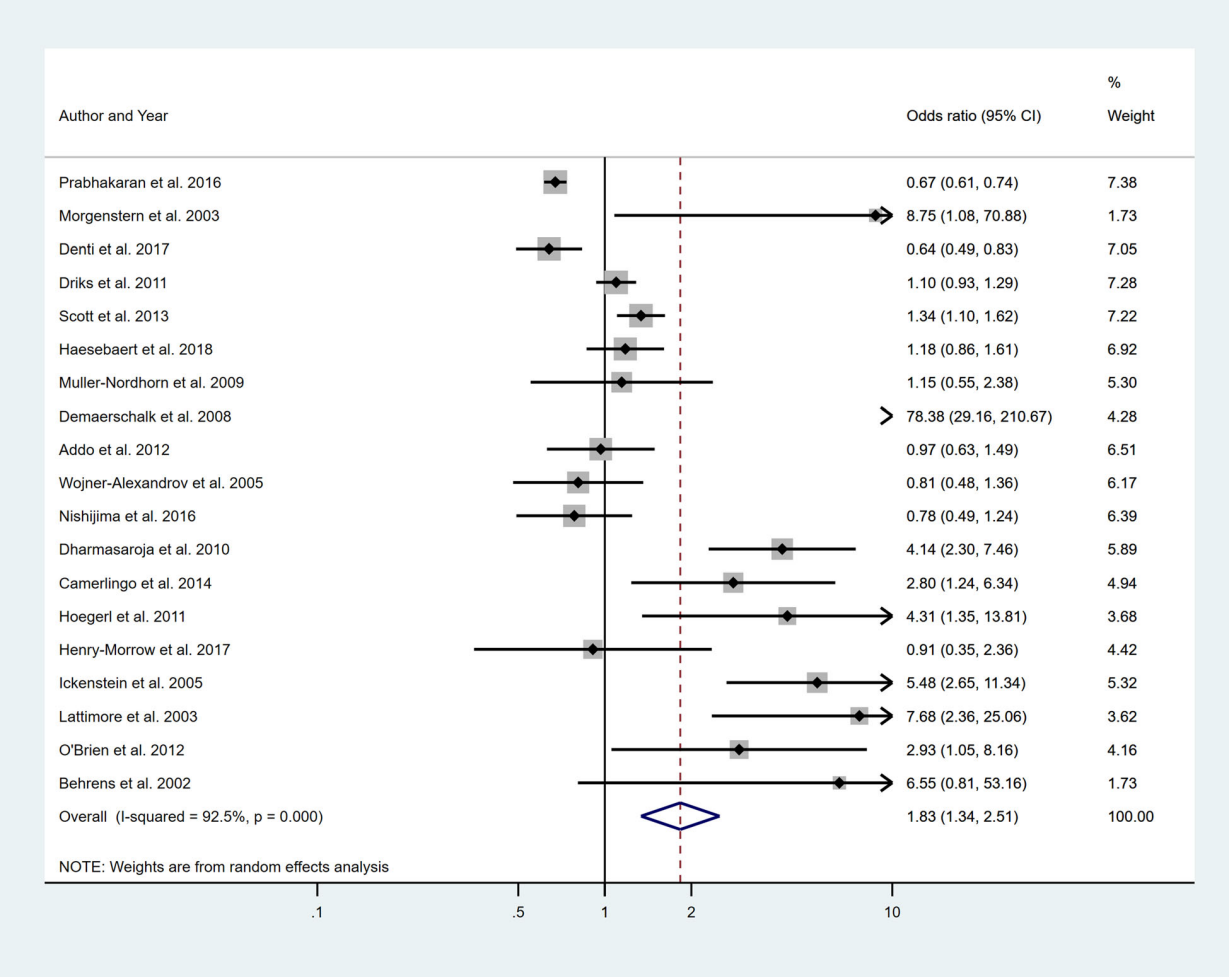
Pooled odds ratio of the intervention effectiveness in the studies that included an education component, at minimum

**Fig. 7:**
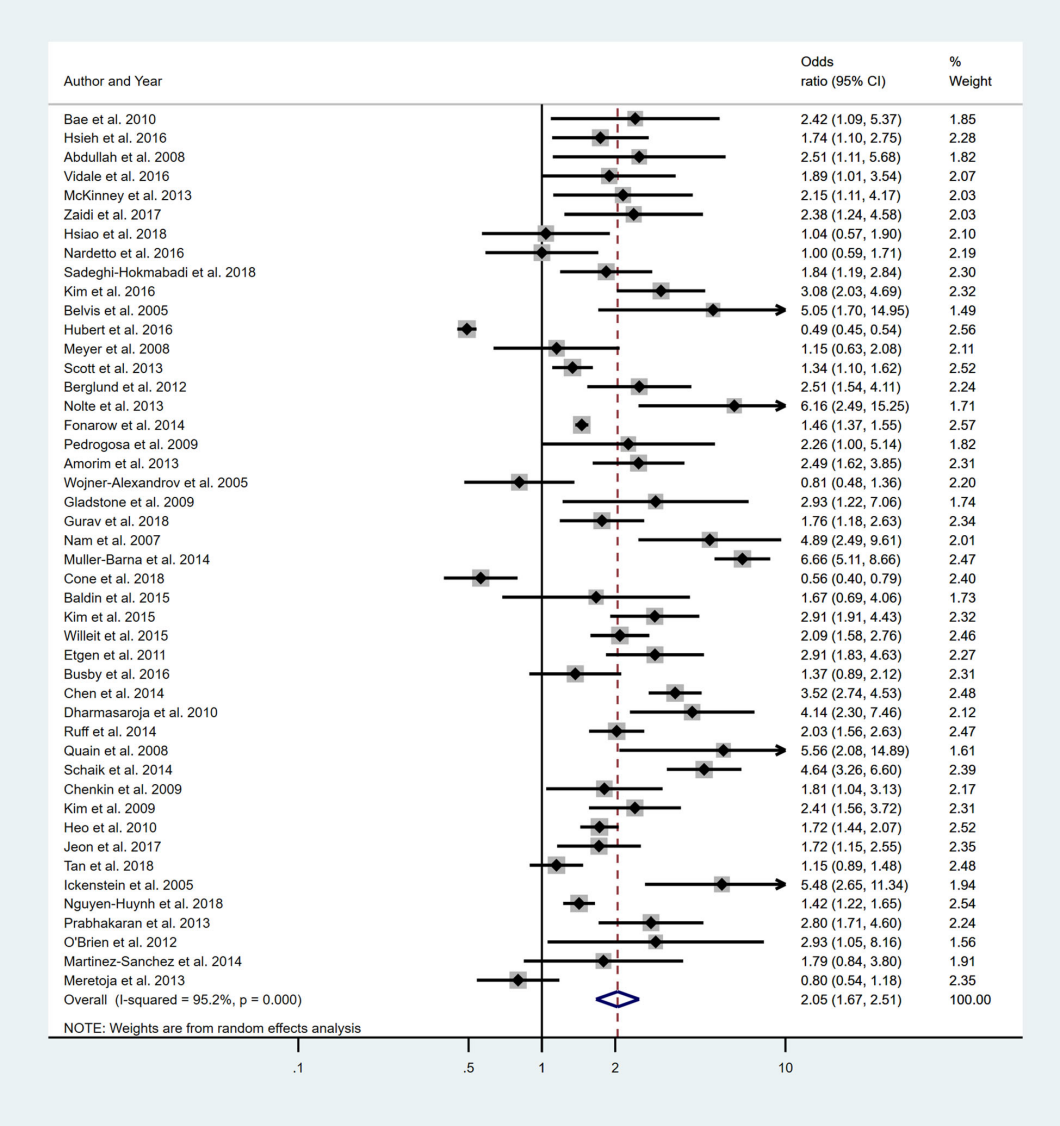
Pooled odds ratio of the intervention effectiveness in the studies that included a persuasion component, at minimum

**Fig. 8:**
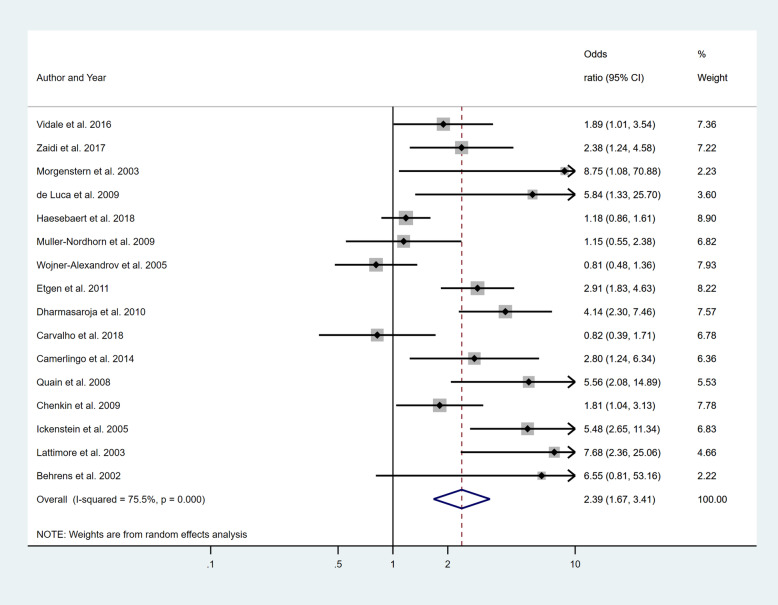
Pooled odds ratio of the intervention effectiveness in the studies that included a Training component, at minimum

**Fig. 9:**
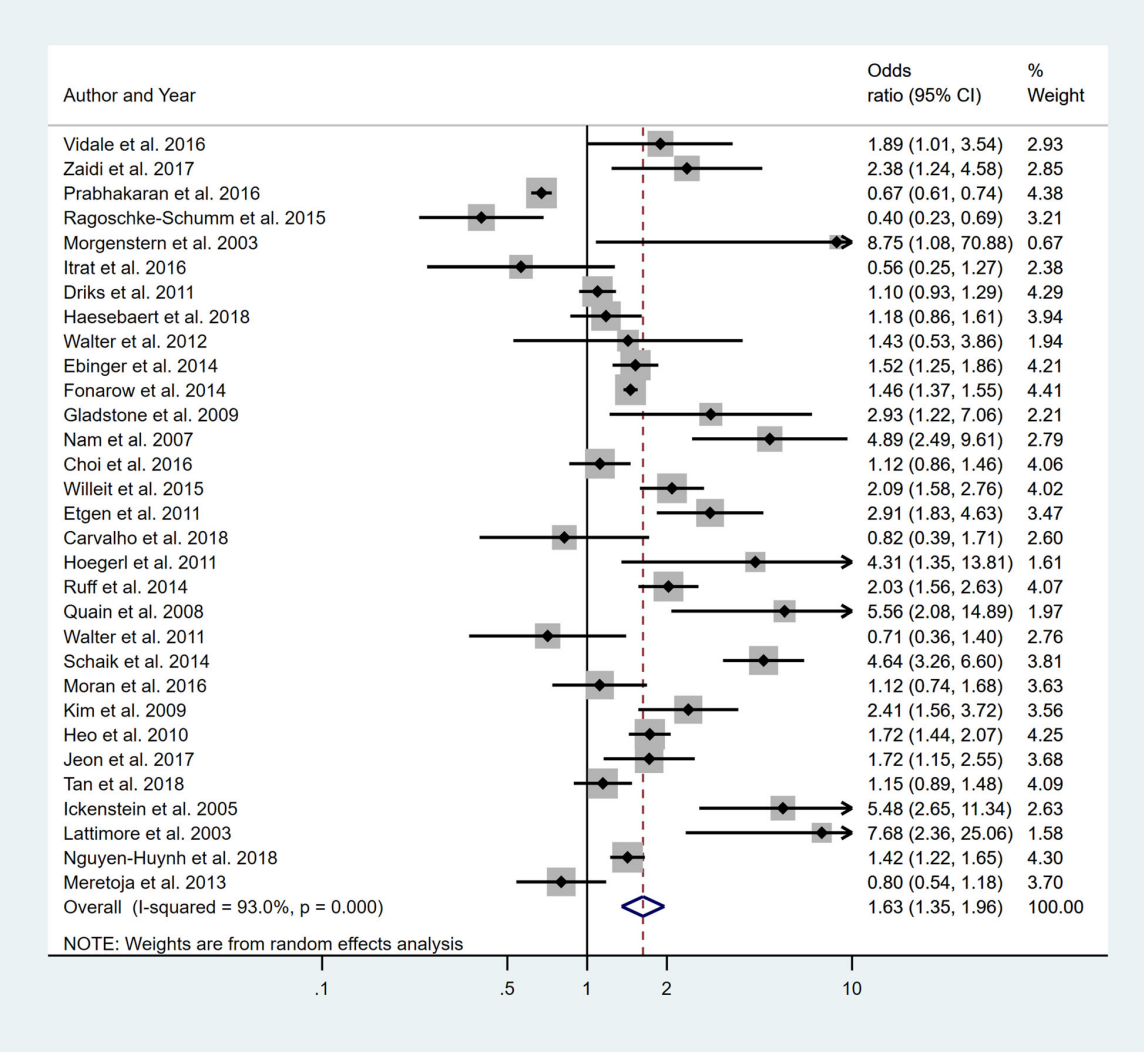
Pooled odds ratio of the intervention effectiveness in the studies that included an Environmental restructuring component, at minimum

**Fig. 10:**
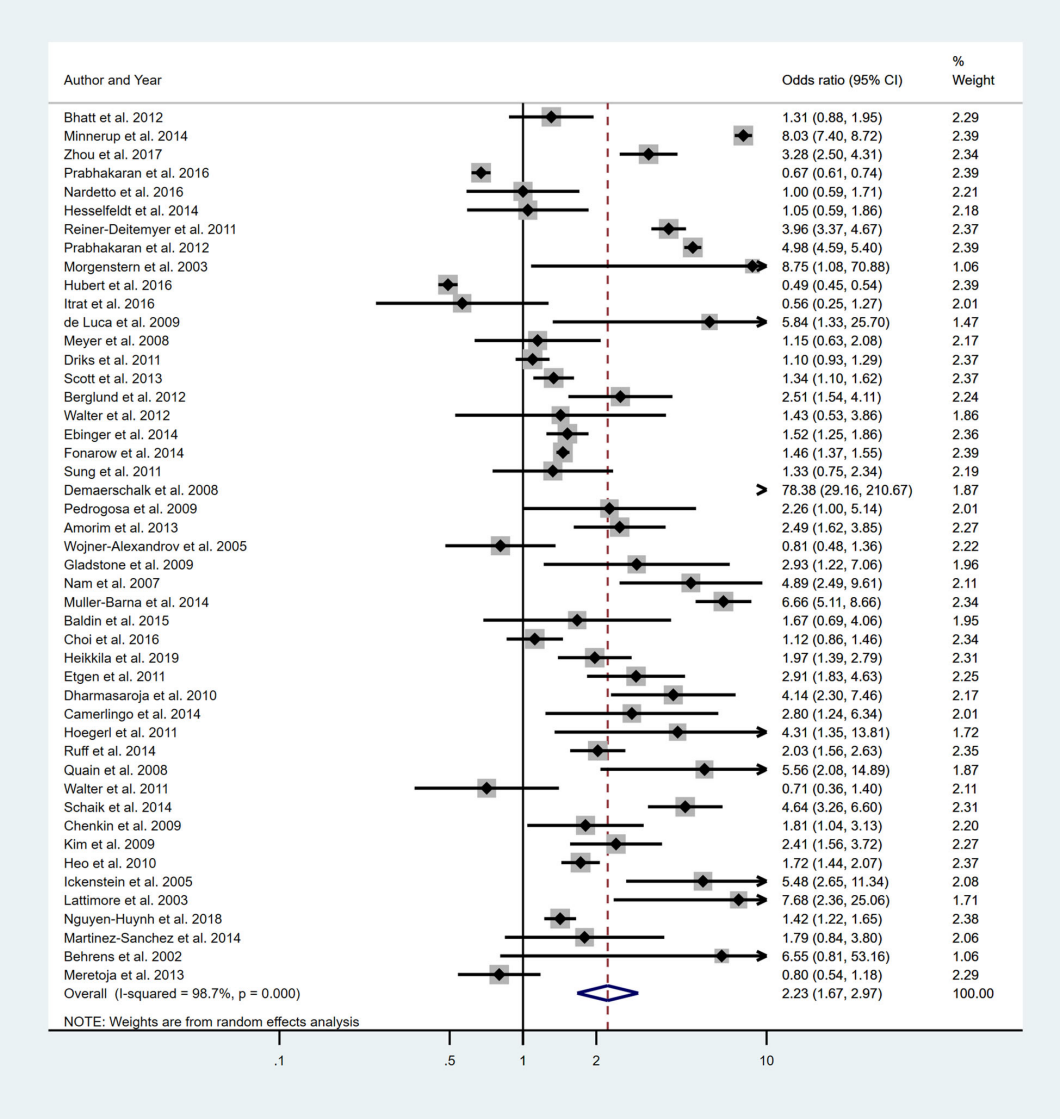
Pooled odds ratio of the intervention effectiveness in the studies that included an Enablement component, at minimum

Five separate meta-analyses (Figs. 6, 7, 8, 9 and 10) were conducted to assess the effect of including at least one of the five BCW intervention types. All five found significant improvement in rates of IVT delivery, with odds ratios of between 1.63 and 2.39 with largely overlapping confidence intervals. High heterogeneity was seen in all meta-analyses (*I*^2^ range 75.5 to 98.8%). Sub-group analyses based on hospital factors addressed by the intervention, denominator used and epidemiological design of the study were again conducted and exhibited moderate to high levels of heterogeneity (*I*^2^). A sensitivity analysis excluding the results of the outlier, Demaerschalk et al. [27] study, was again performed, but this did not substantially change the results.

The meta-regression analysis undertaken to assess whether the number of BCW interventions were associated with efficacy, showed no statistically significant association (OR 1.10; 95% CI 0.93, 1.32, *p* = 0.26).

The contour-enhanced funnel plots investigating the type and number of BCW interventions employed indicated the likelihood of a small-study bias across all meta-analyses, and this is confirmed by Egger’s test (Supplementary file 6, and Supplementary file 7). The missing regions in the contour-enhanced funnel plots indicate that the bias is likely due to a mix of heterogeneity and publication bias. We analysed our results based on various study types (including pre-hospital versus in-hospital) and evaluated the effect on the outcomes. However, the result was the same—an overall significant result with all sub-groups. This result also indicated high heterogeneity and potential publication bias (Supplementary file 8, Supplementary file 9, and Supplementary file 10). Since RCTs provide the strongest evidence, we also looked at the results within the RCT sub-group. In this sub-group, meta-analysis revealed a statistically significant improvement in promoting IVT delivery with an OR of 1.55 (95% CI 1.02–2.35) for the interventions including at least persuasion, OR 1.44 (95% CI 1.04–1.80) for enablement and OR 1.26 (95% CI 1.03–1.54) for environmental restructuring.

## Discussion

The findings of this systematic review and meta-analyses bring together empirical evidence regarding the strategies that are potentially effective in improving IVT rates for AIS. This is the first review to evaluate the differential effect of implementation interventions using a theory-informed (i.e. COM-B) approach using the BCW framework to increase the information available about effective intervrention content. This study found similar overall benefits for the five BCW intervention approaches reviewed. Using the BCW nomenclature, education, persuasion, training, environmental restructuring and enablement all increase thrombolysis uptake with odds ratios of approximately 2. Because the pooled effect sizes were largely overlapping, there seems to be no clear ‘winner’ in terms of which strategy has the largest effect for improving rates of IVT for AIS.

The COM-B theory proposes that multiple aspects of behaviour (capability, opportunity and motivation) are necessary to performing a given behaviour, and the BCW framework proposes multiple factors (education, training, etc.) which support and drive behaviour at an individual and system level. Despite the theory-driven expectation that multiple implementation intervention strategies may potentially increase success in the context of a complex multi-step health care practice, we found no evidence that increasing the number of BCW strategies used in an intervention programme resulted in increased effectiveness. However, the sub-group analysis of RCTs provides some suggestion that persuasion, environmental restructuring and enablement may be particularly effective.

The results should be interpreted with caution based on the existence of both high study heterogeneity or variability of studies, and publication bias. Heterogeneity describes the degree of variability among studies. The presence of high heterogeneity in this study indicates that the true intervention effect may be different in different studies. On the other hand, the presence of bias indicates the possibility of having overestimated the summary effect size [19]. Therefore, until there is more substantial evidence, rather than selecting an intervention approach based on the literature, it may be more relevant to select approaches which best address the major barriers in a given health service context. The findings of the current study align with those of McDermott et al. [4] who concluded that it was difficult to identify any one particular most effective strategy because the overall effects of the identified strategies were almost the same [4]. Conversely, Huang et al. [7] concluded that interventions reducing in-hospital delays may serve as the most effective way to increase IVT delivery; however, several approaches were included under the ‘in-hospital’ approach, which made it difficult to recommend any specific approaches, and they did not find strong evidence to suggest a minimum or maximum number of interventions were required to achieve maximum success.

Although it is unclear why particular strategies were chosen by studies’ authors, it was feasible to categorise the type of intervention strategies used using the BCW theoretical framework and to assign interventions to the categories of education, persuasion, training, environmental restructuring and enablement. This is the only systematic review and meta-analysis that has evaluated the effect of implementation interventions aimed at increasing rates of IVT for AIS using a theoretical framework. However, it should be noted that the studies analysed often did not provide enough detail to unambiguously classify the intervention components based on BCW intervention functions. The inability to find differences in the effect of different BCW intervention functions in improving IVT rates may be due to the fact that the 5 categories are relatively coarse. The BCW intervention functions do lend themselves to finer classification but a lack of detailed information in many of the studies reviewed precluded us using this finer-grained classification. Our study also failed to identify any relationship between the number of intervention components and effectiveness of the intervention. A systematic review on multi-component healthcare interventions indicated that multi-component interventions are difficult to implement and seldom implemented as planned [95]. Complex multi-level multi-component interventions or strategies are more difficult to implement and reproduce in the practical setting with fidelity. Multiple strategies may be needed to engage the variety of professionals involved in care such as IVT, which can increase the level of difficulty when implementing or replicating such interventions [96]. Several studies have showed that poor implementation can reduce intervention impact [97]. Therefore, considering the level of complexity in the implementation process, using multi-component BCW interventions in one package may be challenging.

This study has several strengths and limitations. One limitation, as mentioned above, was that the coding for the BCW intervention functions was based on the information available in the studies’ publications only. Given that the reviewed literature focused on reporting outcome-related aspects of methodology including outcome measures and sample size rather than describing intervention development and content, the literature provides only a very limited understanding of the types of behaviour change interventions that can be effective. A more inter-disciplinary approach to designing these trials may be needed to progress the field. If more explicit descriptions of the intervention used were to be published, this may allow for more in-depth classification and, in turn, more capacity to isolate specific BCW strategies associated with change in AIS practice. It must be acknowledged however, that there is a tension between multi-component and single-focus studies in implementation science. Successful implementation of complex evidence-based practices in any given healthcare setting may require a variety of behaviour change interventions to be implemented. Therefore, evaluating the effectiveness of one BCW intervention function over another might not always be enough to understand how to effect change on a multi-level multi-component process within a complex health system. One strength of the review is the large number of studies which were included and the total of 40,614 IVT cases, thus giving weight to the results. Other strengths include the rigorous review process used to identify studies and extract data, and the use of the BCW theoretical framework for pooling the intervention effects. Our results may assist researchers with the development of future interventions, including avoiding the assumption that using multiple strategies will necessarily increase intervention effectiveness.

In terms of implications for practice in stroke care, this review suggests that despite the complexity of the IVT care pathway, successfully increasing IVT rates does not necessarily require a complex suite of implementation intervention components, nor should it focus on one specific type of intervention function. Therefore, it seems reasonable to suggest that successful interventions will be those that draw on the diversity of potential interventions to address identified challenges in the local context by paying close attention to each aspect of the patient pathway.

## Conclusion

The evidence we provide does not support using one specific type of BCW intervention strategy over another in the setting of IVT implementation and also that using multiple BCW intervention strategies in the same programme may not necessarily increase intervention effectiveness. A more inter-disciplinary approach to study design may be needed. A caveat, however, is that the sub-group analysis with RCTs suggested more effect with persuasion, environmental restructuring and enablement approaches. Our results suggest it may be more relevant for policy makers and implementation scientists to select the approaches that best address the major obstacles in a given context. However, because of the high degree of heterogeneity and publication bias, these conclusions cannot be considered robust.

## Supplementary Information

**Additional file 1.**

**Additional file 2.**

**Additional file 3.**

**Additional file 4.**

**Additional file 5.**

**Additional file 6.**

**Additional file 7.**

**Additional file 8.**

**Additional file 9.**

**Additional file 10.**

## Data Availability

The datasets used and/or analysed during the current study are available from the corresponding author on reasonable request.

## Abbreviations

AIS: Acute ischemic stroke
BCW: Behaviour change wheel
CI: Confidence interval
IVT: Intravenous thrombolysis
OR: Odds ratio
PRISMA: Preferred Reporting Items for Systematic reviews and Meta-analyses
