# Supplementary material for: Effectiveness of interventions to improve rates of intravenous thrombolysis using behaviour change wheel functions: a systematic review and meta-analysis
Source: Implement Sci. 2020 Nov 4;15:98. doi: 10.1186/s13012-020-01054-3 (PMC7641813; doi:10.1186/s13012-020-01054-3)
Supplement: Supplementary file 2 — Additional file 2. [file 13012_2020_1054_MOESM2_ESM.docx]

**Supplement 2:** MEDLINE search strategy and search hits for all databases.

**Database (MEDLINE)**

1 *STROKE/ (318915)

2 "Ischemic stroke".mp. (46405)

3 *Brain Ischemia/ (55531)

4 *Infarction, Middle Cerebral Artery/ (9046)

5 1 or 2 or 3 or 4 (341036)

6 *Tissue Plasminogen Activator/ (23967)

7 tPA.mp. (23060)

8 rtPA.mp. (1201)

9 Alteplase.mp. (2110)

10 Thrombolysis.mp. (26575)

11 6 or 7 or 8 or 9 or 10 (61424)

12 5 and 11 (15260)

13 limit 12 to (English language and year >1995) (12738)

**Database (EMBASE)**

1 Stroke.mp. (457269)

2 "Ischemic Stroke".mp. (80624)

3 "Brain Ischemia".mp. or brain ischemia/ (147119)

4 "Middle Cerebral Artery Infarction".mp. (779)

5 1 or 2 or 3 or 4 (513625)

6 "Tissue Plasminogen Activator".mp. or tissue plasminogen activator/ (36221)

7 tPA.mp. (30399)

8 rtPA.mp. (2471)

9 Alteplase.mp. or alteplase/ (19922)

10 Thrombolysis.mp. (43397)

11 6 or 7 or 8 or 9 or 10 (99184)

12 5 and 11 (31744)

13 limit 12 to (English language and year >1995) (28073)

**Database (PSYCINFO)**

1 Stroke.mp. (35167)

2 "Ischemic Stroke".mp. (5487)

3 "Brain Ischemia".mp. (4311)

4 "Middle Cerebral Artery Infarction".mp. (62)

5 1 or 2 or 3 or 4 (36847)

6 "Tissue Plasminogen Activator".mp. (1011)

7 tPA.mp. (638)

8 rtPA.mp. (136)

9 Alteplase.mp. (170)

10 Thrombolysis.mp. (1115)

11 6 or 7 or 8 or 9 or 10 (1994)

12 5 and 11 (1494)

13 limit 12 to (English language and year >1995) (1396)

**Database (CINAHL)**

| S13 | S5 AND S11 Limiters - Published Date: 19960101-; English Language (6800) |
| --- | --- |
| S12 | S5 AND S11 (6818) |
| S11 | S6 OR S7 OR S8 OR S9 OR S10 (13800) |
| S10 | Thrombolysis (9418) |
| S9 | Alteplase (3682) |
| S8 | rtPA (312) |
| S7 | tPA (4454) |
| S6 | "Tissue Plasminogen Activator" (6188) |
| S5 | S1 OR S2 OR S3 OR S4 (125824) |
| S4 | Middle Cerebral Artery Infarction" (920) |
| S3 | "Brain Ischemia" (10541) |
| S2 | "Ischemic Stroke" (20706) |
| S1 | Stroke (122863) |

**Database (SCOPUS)**

12. ((TITLE-ABS-KEY (Stroke)) OR (TITLE-ABS-KEY ("Ischemic Stroke")) OR (TITLE-ABS-KEY ("Brain Ischemia")) OR (TITLE-ABS-KEY ("Middle Cerebral Artery Infarction"))) AND ((TITLE-ABS-KEY ("Tissue Plasminogen Activator")) OR (TITLE-ABS-KEY (tPA)) OR (TITLE-ABS-KEY(rtPA)) OR ( TITLE-ABS-KEY (Alteplase)) OR ( TITLE-ABS-KEY (Thrombolysis))) (17813)

11. (TITLE-ABS-KEY ("Tissue Plasminogen Activator")) OR (TITLE-ABS-KEY (tPA)) OR (TITLE-ABS-KEY (rtPA)) OR (TITLE-ABS-KEY (Alteplase)) OR (TITLE-ABS-KEY (Thrombolysis)) (91903)

10. TITLE-ABS-KEY (Thrombolysis) AND PUBYEAR > 1995 (32049)

9. TITLE-ABS-KEY (Alteplase) AND PUBYEAR > 1995 (16871)

8. TITLE-ABS-KEY (rtPA) AND PUBYEAR > 1995 (1519)

7. TITLE-ABS-KEY (tPA) AND PUBYEAR > 1995 (33559)

6. TITLE-ABS-KEY ("Tissue Plasminogen Activator") AND PUBYEAR > 1995 (35200)

5. (TITLE-ABS-KEY (Stroke)) OR (TITLE-ABS-KEY ("Ischemic Stroke")) OR (TITLE-ABS-KEY("Brain Ischemia")) OR (TITLE-ABS-KEY ("Middle Cerebral Artery Infarction")) (481645)

4. TITLE-ABS-KEY ("Middle Cerebral Artery Infarction") AND PUBYEAR > 1995 (632)

3. TITLE-ABS-KEY ("Brain Ischemia") AND PUBYEAR > 1995 (116042)

2. TITLE-ABS-KEY ("Ischemic Stroke") AND PUBYEAR > 1995 (53708)

1. TITLE-ABS-KEY (Stroke) AND PUBYEAR > 1995 (427357)
