# Supplementary material for: Effectiveness of interventions to improve rates of intravenous thrombolysis using behaviour change wheel functions: a systematic review and meta-analysis
Source: Implement Sci. 2020 Nov 4;15:98. doi: 10.1186/s13012-020-01054-3 (PMC7641813; doi:10.1186/s13012-020-01054-3)
Supplement: Supplementary file 3 — Additional file 3. [file 13012_2020_1054_MOESM3_ESM.docx]

**Inclusion and Exclusion criteria**

**Title and Abstract**

***Exclusion***

1. Studies not reporting thrombolysis for ischemic stroke.
2. Studies not reporting rates of thrombolysis or onset to needle, onset to door, door to needle, door to imaging or imaging to needle time.
3. Systematic review/Case study/Study protocol.
4. Conference abstract/Poster/Report only.
5. Animal study.
6. Published other than the English language.
7. Published before 1996.

**Full Text**

***Inclusion***

1. Studies investigated the effect of an intervention aimed to improve the rate of thrombolysis/reduce the Onset to Needle, Onset to Door and or Door to Needle time in patients with acute ischemic stroke.
2. Studies have the rates of thrombolysis utilization and /or Onset-to-Door and/or Door-to-Needle time for thrombolysis as their primary outcome.
3. Studies clearly reported the numerator in terms of a number of patients with acute ischemic stroke received intravenous thrombolysis and the denominator in terms of a total number of suspected stroke patients/Diagnosed stroke patients/ Ischemic stroke patients/ Patients eligible for thrombolysis.
4. Study design is experimental studies covering randomized controlled trials and cluster-randomized trials; non-randomized studies such as uncontrolled before-after studies, parallel group trial; and observational studies including cohort, case-control and cross-sectional studies.

***Exclusion***

1. Study findings solely related to hemorrhagic stroke or transient ischemic attack.
2. Studies report rates of thrombolysis other than intravenous thrombolysis only.

**Supplement 3:** Inclusion and Exclusion criteria.
