# Supplementary material for: Effectiveness of interventions to improve rates of intravenous thrombolysis using behaviour change wheel functions: a systematic review and meta-analysis
Source: Implement Sci. 2020 Nov 4;15:98. doi: 10.1186/s13012-020-01054-3 (PMC7641813; doi:10.1186/s13012-020-01054-3)
Supplement: Supplementary file 4 — Additional file 4. [file 13012_2020_1054_MOESM4_ESM.docx]

TR Screened 5,478 Title & Abstract

Included 112 for the full text review

Excluded 5,366 because of:

- 110 for not reporting thrombolysis for AIS
- 5,018 for not reporting thrombolysis rate and or OTN, OTD, DTN time
- 17 systematic review/case studies/protocol
- 221 conference abstract/poster/report

Finally, 207 articles were selected for the full text review

SA Screened 5,478 Title & Abstract

Included 90 for the full text review

Excluded 5,388 because of:

- 119 for not reporting thrombolysis for AIS
- 4,993 for not reporting thrombolysis rate and or OTN, OTD, DTN time
- 21 systematic review/case studies/protocol
- 255 conference abstract/poster/report

MGH Screened 10,956 Title & Abstract

Included 205 for the full text review

Excluded 10,751 because of:

- 220 for not reporting thrombolysis for AIS
- 10,022 for not reporting thrombolysis rate and or OTN, OTD, DTN time
- 32 systematic review/case studies/protocol
- 477 conference abstract/poster/report

10,956 Articles remaining after excluding duplicates

(Title & Abstract screening)

8,961 Duplicates removed

19,917 Records identified from five databases

**Supplement 4:** Study selection process (Full-text).
