# Supplementary material for: Effectiveness of interventions to improve rates of intravenous thrombolysis using behaviour change wheel functions: a systematic review and meta-analysis
Source: Implement Sci. 2020 Nov 4;15:98. doi: 10.1186/s13012-020-01054-3 (PMC7641813; doi:10.1186/s13012-020-01054-3)
Supplement: Supplementary file 5 — Additional file 5. [file 13012_2020_1054_MOESM5_ESM.docx]

207 articles were selected for the full text review

SA Screened 104 Full Texts

Included 45 for data extraction

Excluded 59 because of:

- 29 for not investigating the effect of an intervention aimed to improve thrombolysis rate and or OTN, OTD, DTN time
- 00 for not reporting thrombolysis rate and or OTN, OTD, DTN time as primary outcome
- 14 both mentioned above
- 16 not reporting the numerator and denominator clearly

TR Screened 103 Full Texts

Included 48 for data extraction

Excluded 55 because of:

- 40 for not investigating the effect of an intervention aimed to improve thrombolysis rate and or OTN, OTD, DTN time
- 00 for not reporting thrombolysis rate and or OTN, OTD, DTN time as primary outcome
- 04 both mentioned above
- 11 not reporting the numerator and denominator clearly

MGH Screened 207 Full Texts

Included 75 for data extraction

Excluded 130 because of:

- 83 for not investigating the effect of an intervention aimed to improve thrombolysis rate and or OTN, OTD, DTN time
- 01 for not reporting thrombolysis rate and or OTN, OTD, DTN time as primary outcome
- 20 both mentioned above
- 28 not reporting the numerator and denominator clearly

Finally, 77 articles were selected for the full text review

**Supplement 5:** Study selection process (Included studies).
