# Supplementary material for: Effectiveness of interventions to improve rates of intravenous thrombolysis using behaviour change wheel functions: a systematic review and meta-analysis
Source: Implement Sci. 2020 Nov 4;15:98. doi: 10.1186/s13012-020-01054-3 (PMC7641813; doi:10.1186/s13012-020-01054-3)
Supplement: Supplementary file 6 — Additional file 6. [file 13012_2020_1054_MOESM6_ESM.docx]

Component 1

Component 2

Component 3

Component 4-5

**Supplement 6:** Funnel Plot, Contour enhanced funnel plot, Egger’s test result, based on number of BCW intervention functions.
