# Supplementary material for: Effectiveness of interventions to improve rates of intravenous thrombolysis using behaviour change wheel functions: a systematic review and meta-analysis
Source: Implement Sci. 2020 Nov 4;15:98. doi: 10.1186/s13012-020-01054-3 (PMC7641813; doi:10.1186/s13012-020-01054-3)
Supplement: Supplementary file 7 — Additional file 7. [file 13012_2020_1054_MOESM7_ESM.docx]

Education

Persuasion

Training

Environmental restructuring

Enablement

**Supplement 7:** Funnel Plot, Contour enhanced funnel plot, Egger’s test result, based on type of BCW intervention functions.
