# Supplementary material for: Effectiveness of interventions to improve rates of intravenous thrombolysis using behaviour change wheel functions: a systematic review and meta-analysis
Source: Implement Sci. 2020 Nov 4;15:98. doi: 10.1186/s13012-020-01054-3 (PMC7641813; doi:10.1186/s13012-020-01054-3)
Supplement: Supplementary file 8 — Additional file 8. [file 13012_2020_1054_MOESM8_ESM.docx]

|  | **Number of Studies, n** | **OR (95% CI)** | **Heterogeneity, I^2^ (%)** | **Bias Present** | |
| --- | --- | --- | --- | --- | --- |
|  |  |  |  | **Funnel** | **Contour Enhanced Funnel** |
| ***Uncontrolled Before and After*** | 45 | 2.06 (1.74-2.45) | 90.2 | Yes | Yes |
| Number of Component = 1 | 13 | 1.59 (1.10-2.30) | 89.5 | Yes | Yes |
| Number of Component = 2 | 13 | 2.26 (1.40-3.64) | 93.6 | Yes | Yes |
| Number of Component = 3 | 13 | 2.05 (1.65-2.55) | 85.5 | Yes | Yes |
| Number of Component = 4-5 | 6 | 3.39 (1.70-6.75) | 84.1 | Yes | Yes |
| Addressed Component Education | 12 | 3.09 (1.54-6.16) | 90.5 | Yes | Yes |
| Addressed Component Persuasion | 31 | 2.19 (1.81-2.65) | 91.1 | Yes | Yes |
| Addressed Component Training | 10 | 2.70 (1.64-4.45) | 78.7 | Yes | Yes |
| Addressed Component Environmental Restructuring | 21 | 1.87 (1.56-2.22) | 85.4 | Yes | Yes |
| Addressed Component Enablement | 29 | 2.45 (1.96-3.05) | 91.2 | Yes | Yes |
| ***Parallel Group Trial*** | 22 | 1.85 (1.08-3.18) | 99.3 | Yes | Yes |
| Number of Component = 1 | 15 | 2.29 (1.65-3.19) | 96.4 | Yes | Yes |
| Number of Component = 2 | 3 | 0.63 (0.39-1.03) | 70.0 | - | - |
| Number of Component = 3 | 2 | 2.11 (1.34-3.32) | 0.0 | - | - |
| Number of Component = 4-5 | 2 | 1.94 (0.16-23.1) | 82.6 | - | - |
| Addressed Component Education | 2 | 1.94 (0.16-23.1) | 82.6 | - | - |
| Addressed Component Persuasion | 12 | 1.78 (1.02-3.12) | 94.7 | Yes | Yes |
| Addressed Component Training | 3 | 2.25 (1.45-3.51) | 0.0 | - | - |
| Addressed Component Environmental Restructuring | 6 | 1.04 (0.58-1.87) | 85.2 | Yes | Yes |
| Addressed Component Enablement | 11 | 1.81 (0.83-4.01) | 99.7 | Yes | Yes |
| ***Randomized Controlled Trial*** | 10 | 1.27 (1.00-1.60) | 79.0 | Yes | Yes |
| Number of Component = 1 | 1 | - | - | - | - |
| Number of Component = 2 | 6 | 1.63 (1.20-2.21) | 40.6 | Yes | Yes |
| Number of Component = 3 | 3 | 1.92 (1.05-2.36) | 17.8 | - | - |
| Number of Component = 4-5 | - | - | - | - | - |
| Addressed Component Education | 5 | 1.04 (0.84-1.36) | 80.1 | Yes | Yes |
| Addressed Component Persuasion | 3 | 1.55 (1.02-2.35) | 66.8 | - | - |
| Addressed Component Training | 3 | 1.44 (0.79-2.66) | 53.9 | - | - |
| Addressed Component Environmental Restructuring | 4 | 1.26 (1.03-1.54) | 54.4 | - | - |
| Addressed Component Enablement | 7 | 1.44 (1.04-1.80) | 66.4 | Yes | Yes |

*Bias assesses only when the number of studies in any group are five or more than five.

**Supplement 8:** Sub-group analysis based on epidemiological study design.
