# Supplementary material for: Effectiveness of interventions to improve rates of intravenous thrombolysis using behaviour change wheel functions: a systematic review and meta-analysis
Source: Implement Sci. 2020 Nov 4;15:98. doi: 10.1186/s13012-020-01054-3 (PMC7641813; doi:10.1186/s13012-020-01054-3)
Supplement: Supplementary file 9 — Additional file 9. [file 13012_2020_1054_MOESM9_ESM.docx]

|  | **Number of Studies, n** | **OR (95% CI)** | **Heterogeneity, I^2^ (%)** | **Bias Present** | |
| --- | --- | --- | --- | --- | --- |
|  |  |  |  | **Funnel** | **Contour Enhanced Funnel** |
| ***Pre-Hospital*** | 9 | 1.44 (1.05-1.99) | 95.7 | Yes | Yes |
| Number of Component = 1 | 5 | 1.96 (1.05-2.96) | 97.6 | Yes | Yes |
| Number of Component = 2 | 4 | 1.39 (1.17-1.82) | 47.7 | - | - |
| Number of Component = 3 | - | - | - | - | - |
| Number of Component = 4-5 | - | - | - | - | - |
| Addressed Component Education | 4 | 0.78 (0.61-1.00) | 26 | - | - |
| Addressed Component Persuasion | 3 | 1.54 (1.01-2.12) | 62.9 | - | - |
| Addressed Component Training | 1 | 1.15 (0.55-2.38) | - | - | - |
| Addressed Component Environmental Restructuring | - | - | - | - | - |
| Addressed Component Enablement | 5 | 2.07 (1.27-2.88) | 26 | Yes | Yes |
| ***In-Hospital*** | 35 | 1.84 (1.37-2.48) | 98.4 | Yes | Yes |
| Number of Component = 1 | 10 | 1.94 (1.24-3.04) | 95.5 | Yes | Yes |
| Number of Component = 2 | 12 | 1.53 (0.79-3.00) | 97.3 | Yes | Yes |
| Number of Component = 3 | 7 | 1.45 (1.18-1.79) | 85.1 | Yes | Yes |
| Number of Component = 4-5 | 5 | 3.06 (1.01-9.30) | 96.5 | Yes | Yes |
| Addressed Component Education | 7 | 2.06 (1.29-3.30) | 95.4 | Yes | Yes |
| Addressed Component Persuasion | 21 | 2.06 (1.47-2.89) | 98.9 | Yes | Yes |
| Addressed Component Training | 6 | 3.38 (1.85-6.16) | 73.4 | Yes | Yes |
| Addressed Component Environmental Restructuring | 15 | 1.41 (1.07-1.85) | 95.3 | Yes | Yes |
| Addressed Component Enablement | 27 | 1.93 (1.37-2.71) | 98.7 | Yes | Yes |
| ***Pre- and In-Hospital*** | 33 | 2.41 (1.70-3.41) | 96.6 | Yes | Yes |
| Number of Component = 1 | 13 | 2.14 (1.14-4.00) | 97.1 | Yes | Yes |
| Number of Component = 2 | 6 | 3.18 (1.64-6.17) | 93.1 | Yes | Yes |
| Number of Component = 3 | 11 | 2.16 (1.61-2.88) | 80.5 | Yes | Yes |
| Number of Component = 4-5 | 3 | 2.90 (0.57-14.8) | 86.4 | - | - |
| Addressed Component Education | 8 | 3.27 (1.28-8.34) | 91.1 | Yes | Yes |
| Addressed Component Persuasion | 8 | 4.28 (1.73-10.6) | 97 | Yes | Yes |
| Addressed Component Training | 9 | 1.97 (1.31-2.96) | 67 | Yes | Yes |
| Addressed Component Environmental Restructuring | 13 | 2.05 (1.60-2.64) | 82.4 | Yes | Yes |
| Addressed Component Enablement | 15 | 3.42 (1.98-5.90) | 97.6 | Yes | Yes |

*Bias assesses only when the number of studies in any group are five or more than five.

**Supplement 9:** Sub-group analysis based on before and within hospital study design.
