# Supplementary material for: Effectiveness of interventions to improve rates of intravenous thrombolysis using behaviour change wheel functions: a systematic review and meta-analysis
Source: Implement Sci. 2020 Nov 4;15:98. doi: 10.1186/s13012-020-01054-3 (PMC7641813; doi:10.1186/s13012-020-01054-3)
Supplement: Supplementary file 10 — Additional file 10. [file 13012_2020_1054_MOESM10_ESM.docx]

|  | **Number of Studies, n** | **OR (95% CI)** | **Heterogeneity, I^2^ (%)** | **Bias Present** | |
| --- | --- | --- | --- | --- | --- |
|  |  |  |  | **Funnel** | **Contour Enhanced Funnel** |
| ***Pre-Hospital Notification/Pre-hospital intervention*** | 30 | 1.95 (1.34-2.82) | 96.8 | Yes | Yes |
| Number of Component = 1 | 18 | 1.79 (1.07-3.00) | 98.7 | Yes | Yes |
| Number of Component = 2 | 5 | 1.71 (1.29-2.28) | 29.2 | Yes | Yes |
| Number of Component = 3 | 6 | 2.25 (1.76-2.88) | 0.0 | Yes | Yes |
| Number of Component = 4-5 | 1 | 5.56 (2.08-14.9) | - | - | - |
| Addressed Component Education | 7 | 1.18 (1.03-2.59) | 69.0 | Yes | Yes |
| Addressed Component Persuasion | 18 | 2.16 (1.64-2.84) | 75.7 | Yes | Yes |
| Addressed Component Training | 6 | 2.12 (1.50-3.01) | 32.6 | Yes | Yes |
| Addressed Component Environmental Restructuring | 7 | 2.11 (1.56-2.86) | 48.3 | Yes | Yes |
| Addressed Component Enablement | 12 | 2.55 (1.34-2.82) | 97.0 | Yes | Yes |
| ***In-Hospital Notification*** | 10 | 1.91 (1.39-2.63) | 87.5 | Yes | Yes |
| Number of Component = 1 | 5 | 1.99 (1.26-3.14) | 83.0 | Yes | Yes |
| Number of Component = 2 | 2 | 1.13 (0.94-1.36) | 0.0 | Yes | Yes |
| Number of Component = 3 | 3 | 3.04 (1.33-6.96) | 81.0 | Yes | Yes |
| Number of Component = 4-5 | - | - | - | - | - |
| Addressed Component Education | 1 | 4.32 (1.35-13.8) | - | - | - |
| Addressed Component Persuasion | 7 | 2.07 (1.42-3.01) | 89.1 | Yes | Yes |
| Addressed Component Training | - | - | - | - | - |
| Addressed Component Environmental Restructuring | 5 | 1.76 (1.19-2.60) | 87.4 | Yes | Yes |
| Addressed Component Enablement | 5 | 1.90 (1.24-2.91) | 81.3 | Yes | Yes |
| ***Telemedicine*** | 13 | 1.70 (1.04-3.05) | 97.6 | Yes | Yes |
| Number of Component = 1 | - | - | - | - | - |
| Number of Component = 2 | 10 | 1.41 (1.01-3.24) | 97.7 | Yes | Yes |
| Number of Component = 3 | 1 | 1.42 (1.22-1.65) | - | - | - |
| Number of Component = 4-5 | 2 | 4.63 (2.93-7.31) | 0.0 | - | - |
| Addressed Component Education | 2 | 4.63 (2.93-7.31) | 0.0 | - | - |
| Addressed Component Persuasion | 11 | 1.99 (1.04-3.79) | 98.0 | Yes | Yes |
| Addressed Component Training | 3 | 2.68 (0.89-8.04) | 87.2 | - | - |
| Addressed Component Environmental Restructuring | 4 | 1.40 (0.67-2.93) | 85.2 | - | - |
| Addressed Component Enablement | 12 | 1.80 (1.07-3.33) | 97.8 | Yes | Yes |
| ***Multi-disciplinary Collaboration*** | 15 | 1.70 (1.27-2.29) | 96.1 | Yes | Yes |
| Number of Component = 1 | 2 | 3.86 (2.25-6.62) | 41.5 | - | - |
| Number of Component = 2 | 3 | 2.02 (1.50-2.71) | 25.3 | - | - |
| Number of Component = 3 | 7 | 1.47 (1.12-1.94) | 90.9 | Yes | Yes |
| Number of Component = 4-5 | 3 | 0.85 (0.49-1.48) | 67.7 | - | - |
| Addressed Component Education | 7 | 1.09 (0.78-1.54) | 91.4 | Yes | Yes |
| Addressed Component Persuasion | 8 | 1.72 (1.27-2.32) | 90.3 | Yes | Yes |
| Addressed Component Training | 5 | 1.81 (1.09-3.64) | 68.2 | Yes | Yes |
| Addressed Component Environmental Restructuring | 9 | 2.45 (1.30-4.59) | 97.2 | Yes | Yes |
| Addressed Component Enablement | 11 | 1.60 (1.12-2.28) | 96.9 | Yes | Yes |
| ***Reorganization / increasing capacity*** | 9 | 2.48 (1.28-4.83) | 96.8 | Yes | Yes |
| Number of Component = 1 | 4 | 1.33 (0.41-4.33) | 98.2 | - | - |
| Number of Component = 2 | 2 | 7.35 (0.07-73.1) | 98.3 | - | - |
| Number of Component = 3 | 1 | 2.03 (1.56-2.63) | - | - | - |
| Number of Component = 4-5 | 2 | 4.04 (1.64-9.91) | 55.4 | - | - |
| Addressed Component Education | 2 | 25.1 (2.58-44.1) | 88.5 | - | - |
| Addressed Component Persuasion | 2 | 2.31 (1.64-3.24) | 43.9 | - | - |
| Addressed Component Training | 2 | 4.04 (1.64-9.91) | 55.4 | - | - |
| Addressed Component Environmental Restructuring | 6 | 1.45 (1.07-2.72) | 90.0 | Yes | Yes |
| Addressed Component Enablement | 7 | 3.58 (1.86-6.88) | 95.8 | Yes | Yes |

*Bias assesses only when the number of studies in any group are five or more than five.

**Supplement 10:** Sub-group analysis based on various intervention strategies.
